# Complete Genome Sequence of Bacillus amyloliquefaciens Bacteriophage Ray17

**DOI:** 10.1128/MRA.00134-19

**Published:** 2019-04-11

**Authors:** Ryan Showalter, Imraan Adat, Ronald Raab, Louise Temple

**Affiliations:** a James Madison University, Harrisonburg, Virginia, USA; Loyola University Chicago

## Abstract

Bacteria belonging to the genus Bacillus and their cognate viruses are easily found in the environment. Soil sampled from Rockingham County, VA, yielded the bacteriophage Ray17, which was isolated on Bacillus amyloliquefaciens. Presented here is the complete genome sequence of the unique bacteriophage Ray17 with 43,733 bp and 75 predicted genes.

## Abstract (precis)

Bacteria belonging to the genus Bacillus and their cognate viruses are easily found in the environment. Soil sampled from Rockingham County, VA, yielded the bacteriophage Ray17, which was isolated on Bacillus amyloliquefaciens.

## ANNOUNCEMENT

An isolate of Bacillus amyloliquefaciens was cultured from a soil sample in Rockingham County, VA (global positioning system [GPS] coordinates 38°23′ N 79°03′ W), and identified by 16S rRNA gene sequence analysis. Categorized as a plant growth-promoting rhizobacterium, B. amyloliquefaciens initiates plant growth and produces secondary metabolites that reduce the activity of soilborne plant pathogens (1). Bacteriophage Ray17 was isolated as part of an undergraduate research course using a double-layer agar plate method from the same soil sample (2).

Phage genomic DNA was extracted from 0.5 ml of lysate (>1.0 × 10^9^ PFU/ml) by adding a lysing solution (10 mM EDTA, 2.5% Ficoll-400, 3.3 mM Tris-HCl [pH 8.0], 0.08% SDS) and leaving the mixture at room temperature for 10 min. One milliliter of isopropanol was added, mixed, allowed to sit for 5 min, and centrifuged at 13,000 rpm for 10 min. The pellet was resuspended in 100 μl of sterile water. DNA was sequenced by the North Carolina State Genomic Sciences Laboratory (Raleigh, NC). All methods for sequencing, assembly, and gene predictions were as previously described (3), using default parameters for all programs. Approximately 36,000 high-quality reads randomly derived from 10^6^ reads were assembled into one contig with an average coverage of 120-fold.

**FIG 1:**
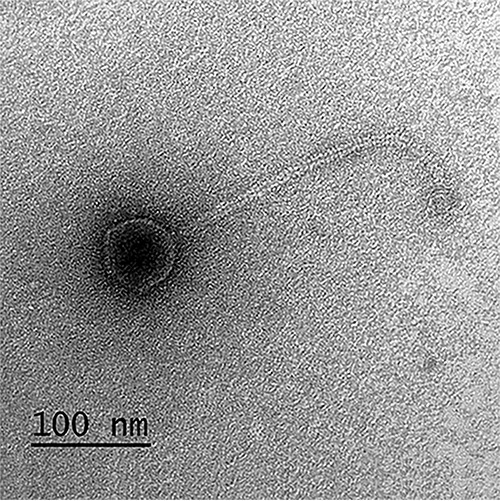
Transmission electron micrograph of Ray17. For electron microscopy, phages from lysate were negatively stained with 1% uranyl acetate on Formvar-coated copper grids and photographed on a Morgagni 268 transmission electron microscope (FEI, Hillsboro, OR).

Ray17 was a siphophage (head, ∼60 nm; tail, ∼285 nm) (Fig. 1). The 43,733-bp genome had a G+C content of 44.55%, correlating closely with the host G+C content of 43.50%. Using PhageTerm, the genome was predicted to be circularly permuted and terminally redundant (4). Whole-genome BLASTn analysis using the nonredundant database (5) revealed that Ray17 was related to the Bacillus subtilis siphovirus SPP1 (6), showing 61% query coverage and 74% identity.

There were 75 genes predicted in the genome of Ray17. Seven of these were structural, such as those encoding head, capsid, and tail proteins. Predicted enzymes included exonuclease, recombinase, and homing endonuclease. No DNA polymerase gene was predicted. In a detailed analysis of SPP1, no DNA polymerase gene was identified (6). Furthermore, host bacterial DNA polymerase was used to reconstitute a replication reaction, indicating that SPP1 may not encode its own DNA polymerase (6). Since the plaque morphology of Ray17 was cloudy and excisionase and recombinase genes were present, it was predicted that Ray17 had a temperate lifestyle. SPP1 has been previously identified as a lytic phage; however, the authors did not indicate whether it was an obligately lytic (virulent) phage (6). No tRNA genes were predicted.

Lysis genes included endolysin and holin genes, in that order. The endolysin was predicted to be a muramidase, which cuts the *N*-acetylmuramic bond of the bacterial peptidoglycan layer (7). The spacing between the endolysin and holin genes strongly suggested a potential secondary structure similar to that of Escherichia coli bacteriophage lambda, indicating that site-directed initiation may be possible in Ray17 (8). There was an additional endolysin gene found in Ray17 that could be a tail lysin; however, it was not found near any other tail structural genes.

The relatively low percent coverage and identity with the most closely related phage, SPP1, determined by using phages catalogued in the Bacillus Phage Database (http://bacillus.phagesdb.org/) for phylogenetic analysis (9), indicate that phage Ray17 may constitute a new cluster of Bacillus phages.

### Data availability.

This whole-genome shotgun project has been deposited in DDBJ/ENA/GenBank under the accession number MH752385. The short-read sequences have been deposited under BioProject number PRJNA517682 and BioSample number SAMN10840650.

## References

[1] Chen XH, Koumoutsi A, Scholz R, Eisenreich A, Schneider K, Heinemeyer I, Morgenstern B, Voss B, Hess WR, Reva O, Junge H, Voigt B, Jungblut PR, Vater J, Süssmuth R, Liesegang H, Strittmatter A, Gottschalk G, Borriss R 2007 Comparative analysis of the complete genome sequence of the plant growth-promoting bacterium *Bacillus amyloliquefaciens* FZB42. Nat Biotechnol 25:1007–1014. doi:10.1038/nbt1325. 17704766

[2] Lorenz L, Lins B, Barrett J, Montgomery A, Trapani S, Schindler A, Christie GE, Cresawn SG, Temple L 2013 Genomic characterization of six novel *Bacillus pumilus* bacteriophages. Virology 444:374–383. doi:10.1016/j.virol.2013.07.004. 23906709

[3] Reveille AM, Eldridge KA, Temple LM 2016 Complete genome sequence of *Bacillus megaterium* bacteriophage Eldridge. Genome Announc 4:e01728-15. doi:10.1128/genomeA.01728-15. 27103735 PMC4841150

[4] Garneau JR, Depardieu F, Fortier LC, Bikard D, Monot M 2017 PhageTerm: a tool for fast and accurate determination of phage termini and packaging mechanism using next-generation sequencing data. Sci Rep 7:8292. doi:10.1038/s41598-017-07910-5. 28811656 PMC5557969

[5] Altschul SF, Gish W, Miller W, Myers EW, Lipman DJ 1990 Basic local alignment search tool. J Mol Biol 215:403–410. doi:10.1016/S0022-2836(05)80360-2. 2231712

[6] Godinho LM, El Sadek Fadel M, Monniot C, Jakutyte L, Auzat I, Labarde A, Djacem K, Oliveira L, Carballido-Lopez R, Ayora S, Tavares P 2018 The revisited genome of *Bacillus subtilis* bacteriophage SPP1. Viruses 10:705. doi:10.3390/v10120705. PMC6316719 30544981

[7] Chapot-Chartier MP 2014 Interactions of the cell-wall glycopolymers of lactic acid bacteria with their bacteriophages. Front Microbiol 5:236. doi:10.3389/fmicb.2014.00236. 24904550 PMC4033162

[8] Raab R, Neal G, Sohaskey C, Smith J, Young R 1988 Dominance in lambda S mutations and evidence for translational control. J Mol Biol 199:95–105. doi:10.1016/0022-2836(88)90381-6. 2965249

[9] Sauder AB, Quinn MR, Brouillette A, Caruso S, Cresawn S, Erill I, Lewis L, Loesser-Casey K, Pate M, Scott C, Stockwell S, Temple L 2016 Genomic characterization and comparison of seven *Myoviridae* bacteriophage infecting *Bacillus thuringiensis*. Virology 489:243–251. doi:10.1016/j.virol.2015.12.012. 26773385

